# Semaglutide improves contractile function in human atrial myocardium of patients with heart failure and preserved ejection fraction

**DOI:** 10.1093/cvr/cvag039

**Published:** 2026-02-03

**Authors:** Philipp Hegner, Maria J Baier, Thomas Krammer, Simon Seitz, Anna-Katharina Käs, Tilman Zschiedrich, David Lukas, Vanessa Lutz, Matthias Wolf, Frederick Sinha, Simon Schopka, Christof Schmid, Kostiantyn Kozakov, Zdenek Provaznik, Lars S Maier, Julian Mustroph, Stefan Wagner

**Affiliations:** Department of Internal Medicine II, University Hospital Regensburg, Regensburg, Germany; Department of Internal Medicine II, University Hospital Regensburg, Regensburg, Germany; Department of Internal Medicine II, University Hospital Regensburg, Regensburg, Germany; Department of Internal Medicine II, University Hospital Regensburg, Regensburg, Germany; Department of Internal Medicine II, University Hospital Regensburg, Regensburg, Germany; Department of Internal Medicine II, University Hospital Regensburg, Regensburg, Germany; Department of Internal Medicine II, University Hospital Regensburg, Regensburg, Germany; Department of Internal Medicine II, University Hospital Regensburg, Regensburg, Germany; Department of Internal Medicine II, University Hospital Regensburg, Regensburg, Germany; Department of Internal Medicine II, University Hospital Regensburg, Regensburg, Germany; Department of Cardiothoracic Surgery, University Hospital Regensburg, Franz-Josef-Strauß-Allee 11, Regensburg 93053, Germany; Department of Cardiothoracic Surgery, University Hospital Regensburg, Franz-Josef-Strauß-Allee 11, Regensburg 93053, Germany; Department of Cardiothoracic Surgery, University Hospital Regensburg, Franz-Josef-Strauß-Allee 11, Regensburg 93053, Germany; Department of Cardiothoracic Surgery, University Hospital Regensburg, Franz-Josef-Strauß-Allee 11, Regensburg 93053, Germany; Department of Internal Medicine II, University Hospital Regensburg, Regensburg, Germany; Department of Internal Medicine II, University Hospital Regensburg, Regensburg, Germany; Department of Pharmacology, University of Regensburg, Regensburg, Germany; Department of Internal Medicine II, University Hospital Regensburg, Regensburg, Germany

**Time of primary review: 22 days**

The GLP-1 receptor agonist semaglutide is an established compound for the treatment of obesity and type 2 diabetes mellitus (T2DM). The recent STEP-HFpEF programme investigated a higher treatment dose of semaglutide in obese patients with heart failure and preserved ejection fraction (HFpEF) and demonstrated an improvement in HF-related symptoms, a reduction of NT-proBNP levels and acute hospitalizations for HF.^1^ The mechanisms underlying the functional cardiac improvement are not understood but exceed a simple weight reduction effect.^1^ The magnitude of improvement in HF symptoms was also superior in patients with AF at baseline.^2^ Moreover, patients with higher NT-proBNP at baseline exhibited more pronounced improvement in HF symptoms despite similar weight loss.^3^ Atrial cardiomyopathy is characterized by structural remodelling and a reduction of atrial contractility and substantially contributes to morbidity and mortality in patients with HFpEF.^4^

In human atrial myocardium, the GLP-1 receptor is expressed to a similar extent as in pancreatic tissue.^5^ Thus, the reduction of NTproBNP observed in the STEP-HFpEF programme may point towards direct atrial effects of semaglutide possibly mediated via the GLP-1 receptor. Previously, we have demonstrated cardioprotective effects of semaglutide in ventricular tissue of patients with heart failure including patients with HFrEF, mediated via a reduction in proarrhythmogenic late sodium current and increase in sarcoplasmic reticulum Ca content similar to CaMKII inhibition, while no effects were observed in non-failing ventricular myocardium, but the efficacy in atrial myocardium of HFpEF patients remained unclear.^6^ Therefore, the purpose of this translational study was to investigate possible direct effects of semaglutide on atrial function with a focus on isolated human atrial cardiomyocytes and multicellular tissue preparations in a cohort with predominantly HFpEF patients and correlate results with clinical data.

Experiments were performed in compliance with the Declaration of Helsinki, approved by the local ethics committee (University Regensburg, Bavaria, Germany; 22-2802-101), and patients gave prior written informed consent. Baseline patient data of 85 patients undergoing CABG and/or valve surgery for which intraoperative right atrial myocardial biopsies were obtained across all experiments is presented in *Figure 1A*, a total of 57% fulfilled the diagnostic criteria for HFpEF according to current ESC guidelines, and NTproBNP levels were similar to recent HFpEF studies. Analysis of human atrial tissue, atrial slices and atrial cardiomyocytes was performed as described previously.^6^ Data are expressed as mean ± standard error of the mean per patient unless noted otherwise. Statistical comparisons were performed using Graph Pad Prism 10 and the appropriate tests are indicated in the figure legend. Two-sided *P* < 0.05 was considered significant.

**Figure 1:**
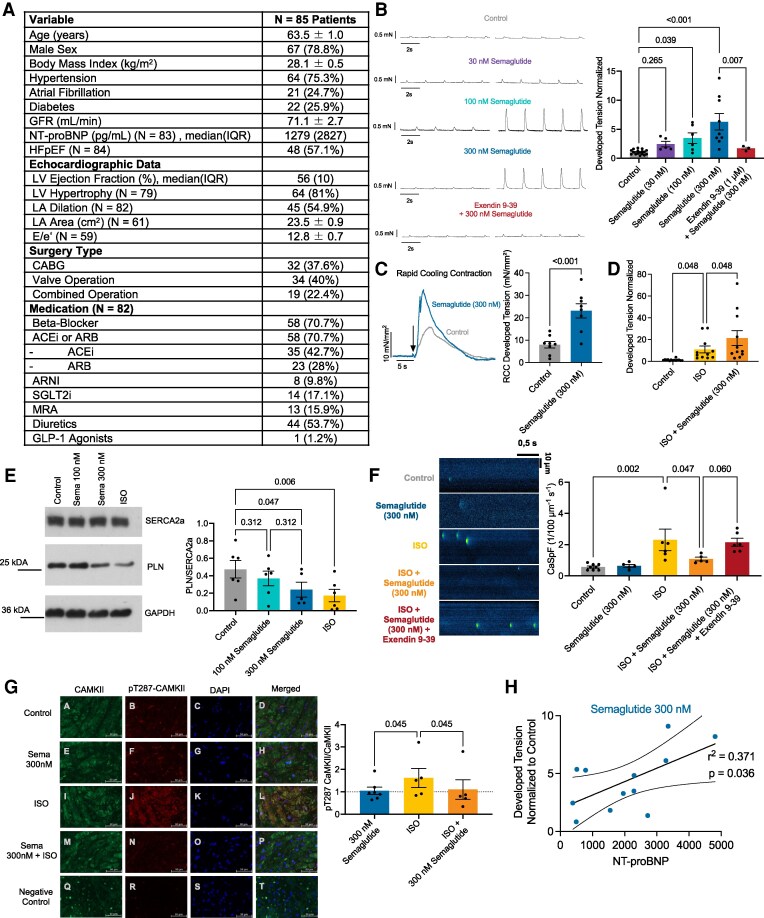
(*A*) Clinical characteristics of patients for which atrial tissue was analysed. (*B*) Original recordings of atrial slices electrically stimulated (0.5 Hz) (left panel); and exposed to control (*N* = 17 patients), semaglutide 30 nM (*N* = 5), 100 nM (*N* = 6), 300 nM (*N* = 9), 300 nM + exendin 9–39 (*N* = 3) developed tension data per patient (right panel), repeated-measures (RM) mixed-effects analysis with Holm–Sidak post-hoc test. (*C*) Rapid cooling contractions elicited in atrial trabeculae (left panel), developed tension per patient (right panel), control (*N* = 8 patients), semaglutide 300 nM (*N* = 8), paired *t*-test. (*D*) Developed tension per patient in atrial slices exposed to control (*N* = 14), isoprenaline 100 nM (ISO) (*N* = 11), and ISO + semaglutide 300 nM (*N* = 11), RM-mixed-effects-analysis with Holm–Sidak post-hoc test. (*E*) Western Blot original gel images (left) and corresponding densitometric data per patient for PLN/SERCA2a expression ratio (right) for atrial myocardium incubated with control (*N* = 6 patients), semaglutide 100 nM (*N* = 6), 300 nM (*N* = 5), and ISO (*N* = 6), RM-mixed-effects-analysis with Holm-Sidak post-hoc test. (*F*) Ca-sparks recorded in Fluo-4-AM loaded atrial cardiomyocytes (left panel—original line scans), Ca-spark frequency data per patient (right panel) control (*N* = 8 patients/22 cells), semaglutide 300 nM (*N* = 5/12), ISO (*N* = 6/25), ISO + semaglutide (*N* = 5/17), ISO + semaglutide + exendin-9–39 (*N* = 6/19), RM-mixed-effects-analysis with Holm-Sidak post-hoc test. (*G*) Left panel—Original immunohistochemistry staining of CaMKII and pT287-CaMKII in human atrial myocardium; right panel—pT287-CaMKII/CaMKII intensity ratio normalized to control per patient exposed to semaglutide 300 nM (*N* = 7 patients), ISO (*N* = 5), ISO + semaglutide (*N* = 5), RM-mixed-effects analysis with Holm-Sidak post-hoc test. (*H*) Linear regression analysis of patient NTproBNP levels vs. atrial trabeculae developed tension exposed to semaglutide 300 nM as fold of control, (*N* = 12 patients).

Isolated human right atrial trabeculae and myocardial slices (original traces in *Figure 1B*, left) were exposed to semaglutide (30–300 nM) at pacing rates (0.5 Hz, 1, 2, and 4 Hz not depicted). Acute exposure resulted in a significant, concentration-dependent increase in developed tension, with no effect at 30 nM, and marked increases at 100 and 300 nM (*Figure 1B*, right). The inotropic response was abrogated by GLP-1 receptor antagonism (Exendin 9–39), confirming receptor dependence (*Figure 1B*, right). Mechanistically, semaglutide significantly increased SR Ca load, as measured by rapid cooling contractures elicited in trabeculae by rapid superfusion with chilled solution (2°C) (*Figure 1C*). In contrast, blockade of L-type Ca channels with nifedipine did not affect semaglutide-induced improvement of contractility, suggesting a distinct mechanism from classical β-adrenergic stimulation (not shown). Instead, semaglutide further augmented the inotropic response to isoproterenol (ISO), doubling the contractility achieved with ISO alone (*Figure 1D*). Western blot analysis revealed a reduced (pentameric) phospholamban (PLN)/SERCA2a ratio, indicating enhanced SERCA2a-mediated Ca reuptake (*Figure 1E*). SR Ca load is strongly connected to diastolic SR Ca leak, and SR Ca leak can lead to cellular arrhythmias due to induction of a proarrhythmogenic transient inward current I_Ti_ by resulting electrogenic Na/Ca exchanger transport and also Ca-induced Ca release.^7^ Importantly, semaglutide did not increase the incidence of spontaneous arrhythmias, either alone or with ISO (*P* = n.s., not shown).

The acute positive inotropic effect of beta-adrenergic stimulation is mediated via a cAMP second messenger pathway. On the other hand, stimulation of the GLP-1R could also increase cAMP levels. Only at high concentrations (300 nM), semaglutide increased bulk cAMP levels comparable to ISO (*P* = 0.048 vs. control, not shown). However, in contrast to ISO, semaglutide suppressed ISO-induced SR Ca leak, assessed via Ca spark frequency in isolated Fluo-4-AM loaded atrial cardiomyocytes in a GLP-1R dependent manner (*Figure 1F*). An attenuation of ISO-induced effects was also previously observed in ventricular murine cardiomyocytes exposed to supratherapeutic semaglutide concentration.^8^

The cardiac stress kinase CaMKII is crucially involved in Ca homeostasis and can induce diastolic SR Ca leak (via RyR2 phosphorylation), thereby depleting the SR Ca content, reducing contractility and increasing arrhythmias.^9^ While ISO is known to activate CaMKII resulting in increased autophosphorylation, a surrogate for CaMKII activity,^10^ semaglutide alone did not, and co-treatment attenuated ISO-induced CaMKII T-287 autophosphorylation (*Figure 1G*). These results suggest semaglutide may enhance contractility through increased SR Ca load and SERCA2a function, while avoiding pro-arrhythmogenic CaMKII activation and SR Ca leak. However, no CaMKII activation or arrhythmogenesis was observed, implying compartmentalized or downstream differences in GLP-1R vs. β-adrenergic receptor signalling pathways. This divergence may explain the beneficial safety characteristics of semaglutide despite enhanced inotropy.

Finally, the magnitude of the atrial inotropic response correlated with patient NT-proBNP levels (*Figure 1H*) but not with LVEF (not shown). This suggests that atrial-selective effects depend on receptor availability and pathological remodelling and underscore effects observed in the clinical studies including independence from LVEF.

Our findings provide mechanistic support for the early and weight-loss independent improvement in HF symptoms observed in STEP-HFpEF. The concentration range (100–300 nM) used is close to pharmacokinetic data of clinically relevant high-dose regimens (≥2.4 mg weekly), since plasma concentrations of ∼120 nM were reported in patients receiving high-dose semaglutide. These data support a direct myocardial effect of semaglutide, particularly in atrial tissue affected by remodelling and dysfunction, but patient heterogeneity, influence of medications and possible paracrine effects may contribute. In summary, semaglutide induces a GLP-1R-dependent increase in atrial contractility through enhanced SR Ca handling and reduced PLN/SERCA2a ratio, without promoting arrhythmias and CaMKII activation. These findings support further investigation into semaglutide as a therapeutic agent in HFpEF.

## Data Availability

The original contributions presented in the study are included in the article, further inquiries can be directed to the corresponding author.
